# Characterization of the microbiota in the guts of *Triatoma brasiliensis* and *Triatoma pseudomaculata* infected by *Trypanosoma cruzi* in natural conditions using culture independent methods

**DOI:** 10.1186/s13071-015-0836-z

**Published:** 2015-04-24

**Authors:** Marcia Gumiel, Fabio Faria da Mota, Vanessa de Sousa Rizzo, Otília Sarquis, Daniele Pereira de Castro, Marli Maria Lima, Eloi de Souza Garcia, Nicolas Carels, Patricia Azambuja

**Affiliations:** Laboratório de Bioquímica e Fisiologia de Insetos, Instituto Oswaldo Cruz, Fundação Oswaldo Cruz (IOC/FIOCRUZ), Rio de Janeiro, RJ Brazil; Laboratório de Biologia Computacional e Sistemas, Instituto Oswaldo Cruz, Fundação Oswaldo Cruz (IOC/FIOCRUZ), Rio de Janeiro, RJ Brazil; Laboratório de Ecoepidemiologia da Doença de Chagas, Instituto Oswaldo Cruz, Fundação Oswaldo Cruz (IOC/FIOCRUZ), Rio de Janeiro, RJ Brazil; Laboratório de Modelagem de Sistemas Biológicos, National Institute for Science and Technology on Innovation in Neglected Diseases (INCT-IDN), Centro de Desenvolvimento Tecnológico em Saúde (CDTS), Fundação Oswaldo Cruz (FIOCRUZ), Rio de Janeiro, RJ Brazil; Departamento de Entomologia Molecular, Instituto Nacional de Entomologia Molecular (INCT-EM), Rio de Janeiro, RJ Brazil

**Keywords:** *Triatoma brasiliensis*, *Triatoma pseudomaculata*, Peridomestic habitats, Gut bacteria, *Trypanosoma cruzi*, COI barcoding, DGGE, PCR, Cloning, Pyrosequencing

## Abstract

**Background:**

Chagas disease is caused by *Trypanosoma cruzi*, which is transmitted by triatomine vectors. The northeastern region of Brazil is endemic for Chagas disease and has the largest diversity of triatomine species. *T. cruzi* development in its triatomine vector depends on diverse factors, including the composition of bacterial gut microbiota.

**Methods:**

We characterized the triatomines captured in the municipality of Russas (Ceará) by sequencing the cytochrome c oxidase subunit I (COI) gene. The composition of the bacterial community in the gut of peridomestic *Triatoma brasiliensis* and *Triatoma pseudomaculata* was investigated using culture independent methods based on the amplification of the 16S rRNA gene by polymerase chain reaction (PCR), denaturing gradient gel electrophoresis (DGGE), DNA fragment cloning, Sanger sequencing and 454 pyrosequencing. Additionally, we identified TcI and TcII types of *T. cruzi* by sequencing amplicons from the gut metagenomic DNA with primers for the mini-exon gene.

**Results:**

Triatomines collected in the peridomestic ecotopes were diagnosed as *T. pseudomaculata* and *T. brasiliensis* by comparing their COI sequence with GenBank. The rate of infection by *T. cruzi* in adult triatomines reached 80% for *T. pseudomaculata* and 90% for *T. brasiliensis*. According to the DNA sequences from the DGGE bands, the triatomine gut microbiota was primarily composed of Proteobacteria and Actinobacteria. However, Firmicutes and Bacteroidetes were also detected, although in much lower proportions. *Serratia* was the main genus, as it was encountered in all samples analyzed by DGGE and 454 pyrosequencing. Members of Corynebacterinae, a suborder of the Actinomycetales, formed the next most important group. The cloning and sequencing of full-length 16S rRNA genes confirmed the presence of *Serratia marcescens*, *Dietzia* sp., *Gordonia terrae*, *Corynebacterium stationis* and *Corynebacterium glutamicum*.

**Conclusions:**

The study of the bacterial microbiota in the triatomine gut has gained increased attention because of the possible role it may play in the epidemiology of Chagas disease by competing with *T. cruzi*. Culture independent methods have shown that the bacterial composition of the microbiota in the guts of peridomestic triatomines is made up by only few bacterial species.

**Electronic supplementary material:**

The online version of this article (doi:10.1186/s13071-015-0836-z) contains supplementary material, which is available to authorized users.

## Background

Chagas disease remains a serious health concern in developing countries, with approximately 8 million people living in the chronic phase of this parasitosis [1]. *Trypanosoma cruzi,* the causative agent, is mainly transmitted to humans by bugs from the Triatominae family distributed along the American continent.

*Triatoma brasiliensis* and *Triatoma pseudomaculata* are two species of triatomines mainly found in the northeastern region of Brazil, which is considered the epicenter of their dispersion [2,3] and is ranked as the largest region in triatomine diversity [4]. These two vector species show eclectic behavior in relation to the ecotopes where they are found (sylvatic, domestic and peridomestic), raising the question of their respective epidemiological importance in the transmission of *T. cruzi* to humans [5]. In the *Caatinga*, a bioma of northeastern Brazil that is characterized by a semi-arid climate with shrub and tree coverage lower than 60%, *T. brasiliensis* is commonly found in domestic and peridomestic ecotopes. Its natural microhabitat composed of wood piles, cacti, timber piles, and stones is frequently occupied by rodents [5-7], but it is also associated with the habitats of peridomestic animals (dogs, chickens, goats) often diagnosed with *T. cruzi* infections [8]. The coexistence of dogs in these rural areas serves as alternative reservoir with close contact to humans, making peridomestic habitats an interface with specific features compared to the habitats in the wild and domestic areas. Because of these specificities, triatomine vectors in peridomestic habitats may house microbiota different from the wild habitat, which may have epidemiological consequences for Chagas disease [8].

In addition to insect vectors, *T. cruzi* can also be isolated from a wide range of mammalian sylvatic hosts, domestic animals and humans. These isolates showed different infectivity, pathogenicity, and virulence rates [9,10]. The considerable genetic and phenotypic variation of *T. cruzi* strains led us to classify them into different groups. *T. cruzi* classification can be made using specific primers based on mini-exon (TcI, TcII and Z3) [11]. More recently, the *T. cruzi* classification by molecular markers was separated into six Discrete Typing Units (DTUs) with TcI and TcII being the main groups, and TcII being itself divided into five subtypes identified as “a” to “e” [12]. This classification is now simply referred to as TcI to TcVI [13-15].

Another aspect of parasite infection in triatomine vectors that requires attention is the interaction between *T. cruzi* and the intestinal microbiota of triatomine vectors. There is evidence that the species composition spectrum of bacterial communities can affect the vector competence for parasite transmission [16]. A protective role for midgut bacteria against *T. cruzi* was demonstrated *in vitro* [17,18] as well as *in vivo* using *Rhodnius prolixus* as a model organism [19]. In the latter, the use of antibiotics to clear gut microbiota supported the development of *T. cruzi* [19].

There are still few studies of the taxonomic characterization of gut microbiota in triatomines. Most are based on culture-dependent methods [20-22]. Approaches based on microbiota cultivation in controlled conditions are estimated to allow the recovery of a limited number of bacterial species in nature [23,24]. Therefore, this method is expected to introduce a bias in the estimation of the real species composition of microbial communities because of the changes induced by the culture process itself [25,26].

By contrast, approaches based on the analysis of metagenomic DNA in triatomine guts may better reflect the actual proportion and nature of microbial species. The fingerprinting of a bacterial community can be obtained for an individual insect gut by amplification with polymerase chain reaction (PCR) of 16S rDNA, cloning and sequencing or, alternatively, by a gel separation of PCR amplicons using denaturing gradient gel electrophoresis (DGGE) prior to the sequencing step. Recently, the DNA next generation sequencing (NGS) technologies such as 454 Roche pyrosequencing have been applied to the deep sequencing of a limited number of human gut microbiota, resulting in thousands of ribosomal sequences of bacterial communities [27].

The metagenomic DNA of triatomine guts also contain DNA of protozoan parasites that could be used to confirm the presence of *T. cruzi*. Moreover, gut metagenomic DNA can also be used to assess a molecular taxonomic identification of triatomine species based on mitochondrial genes such as cytochrome c oxidase I (COI) and cytochrome B [28,29]. It is particularly important to avoid the wrong identifications that occur when vector specimens come from areas where two or more endemic species share morphological characteristics, as in the northeastern region of Brazil [6].

In this study, we characterized and compared the bacterial microbiota composition of the gut of *Triatoma* spp. kept in the insectary to that of specimens taken from peridomestic habitats in an endemic area in the northeastern region of Brazil. For these purposes, we used PCR amplification, denaturing gradient gel electrophoresis (DGGE), 16S rDNA fragment cloning, Sanger sequencing and 454 pyrosequencing of the 16S rRNA gene. In addition, we report the occurrence of TcI and TcII types of *T. cruzi* through the PCR amplification of the mini-exon gene in the gut of peridomestic *T. brasiliensis* and *T. pseudomaculata*.

## Methods

### Ethics statement

The animals used as feeding sources to maintain triatomines at FIOCRUZ were treated according to the Ethical Principles in Animal Experimentation approved by the Ethics Committee in Animal Experimentation (CEUA/FIOCRUZ) under the license numbers LW-24/2013 and following the protocol from *Conselho Nacional de Experimentação Animal/Ministério de Ciência e Tecnologia*. Triatomines were captured under the license L14323-7 given by the *Sistema de Autorização e Informação em Biodiversidade* (SISBIO) of the *Instituto Chico Mendes de Conservação da Biodiversidade/Ministério do Meio Ambiente* (MMA).

### Triatomines reared in the insectary

*Triatoma* spp*.* specimens were taken from the insectary of the *Laboratório de Ecoepidemiologia da Doença de Chagas* (*Instituto Oswaldo Cruz, Fundação Oswaldo Cruz*) according to the age of their colony *i.e.*, (i) a few weeks, (ii) three years and (iii) five years. These triatomines were fed on living mice (*Mus musculus*) in the laboratory until the analysis of their gut microbiota.

### Morphological identification of triatomines captured in peridomestic habitats

Thirty three specimens of triatomines were captured in 2012 in the municipality of Russas (Ceará) located in the Jaguaribe River Valley in the northeastern region of Brazil (4°56′25″S and 37°58′33″W). The climate of this region is tropical, hot and semi-arid corresponding to the *Caatinga* bioma. The triatomine specimens were transported alive in plastic tubes to the laboratory and morphologically characterized for their taxonomic status according to Lent and Wygodzinsky [30].

### Specimen dissection

Insects were surface sterilized with 0.5% sodium hypochlorite solution and rinsed three times in sterile distilled water before dissection. Triatomines (nymph and adult stages) were dissected by cutting the connective membrane laterally and taken out of the dorsal cuticle over sterile glass slides with sterilized forceps and disposable needles using a stereoscopic microscope model Motic Q766 (Quimis, Diadema, SP, Brazil) at 12x magnification. Afterwards, the gut samples (from proventriculus to rectum) were collected in sterile Eppendorf tubes and maintained at −80°C until use. All steps were performed under aseptic conditions.

### Metagenomic DNA extraction

We extracted metagenomic DNA from the gut samples with the commercial Fast-DNA® Spin Kit for Soil (Qbiogene, CA, USA). The gut samples were added to Lysis Matrix E tubes (BIO101 Systems, Qbiogene) and run twice in a Mini Beadbeater-96 high-throughput cell disrupter (Biospec Products, Bartlesville, OK, USA) for 45 s at a speed level of 5.5. The extraction was then carried out according to the manufacturer’s instructions. DNA extracts were visualized on 1% (w/v) agarose gels to assess their integrity and purity.

### Molecular identification of *Triatoma* specimens collected in peridomestic areas

The precise phylogenetic position of triatomines was obtained by reference to the COI gene. COI was PCR amplified as described by Folmer et al. [28]. The 709 bp amplicons were obtained by using the forward primer LCO1490 (5′–GGT CAA CAA ATC ATA AAG ATA TTG-3′) and the reverse primer HCO2198 (5′- TAA ACT TCA GGG TGA CCA AAA AAT CA-3′). PCR amplifications were carried out with approximately 50 ng DNA template, 1X reaction buffer, 0.5 mM MgCl_2_, 0.1 μM dNTP, 0.2 μM of each primer, 0.05 U GoTaq DNA polymerase (Promega, USA) and water to a final volume of 25 μl.

Negative controls with ultrapure water for molecular biology (Promega, USA) were routinely included to check contamination. PCR conditions were an initial 94°C step for 3 min, a cycle of denaturation at 94°C for 30 s, annealing at 45°C for 30 s and extension at 72°C for 1 min was performed 5 times and repeated another 35 times, with a second annealing temperature at 51°C for 1 min followed by a 10 min at 72°C final extension. Amplicons were analyzed by agarose gel (1.5%) electrophoresis and visualized under ultraviolet light after ethidium bromide staining. The products were sequenced with an ABI Prism 3730 XL sequencer (Applied Biosystems). Sequences were compared to GenBank for similarity search using BLASTN [31]. The best hit sequences were then aligned via ClustalW2 [32] and checked for consistency in Bioedit [33]. A phylogenetic tree based on maximum likelihood following the GTR model with 1,000 bootstrap replicates was constructed using Mega v.5.01 [34].

### *T. cruzi* detection by mini-exon amplification from metagenomic DNA

*T. cruzi* (TcI and TcII) from the triatomines of peridomestic habitats was diagnosed by singleplex PCR on gut metagenomic DNA samples using the ME (5′-TAC CAA TAT AGT ACA GAA ACT G-3′) and TcI (5′-ACA CTT TCT GTG GCG CTG ATC G-3′) or TcII (5′-TTG CTC GCA CAC TCG GCT GCA T-3′) specific primers. The ME primer was originally designed to amplify mini-exons and is specific to *T. cruzi* [11,35]. DM28c and Y strains were used as positive controls of the TcI and TcII groups, respectively. PCR amplifications were carried out containing approximately 50 ng DNA template, 1X reaction buffer, 0.2 mM MgCl_2_, 0.2 μM dNTP, 0.28 μM of each primer, 0.0125 U GoTaq DNA polymerase (Promega, USA) and water to a final volume of 25 μl. The negative controls for the TcI and TcII PCR amplifications were the buffer without target genomic DNA. The PCR conditions used were a starting step at 95°C for 1 min followed by 35 cycles of denaturation at 94°C for 30 s, annealing at 57°C for TcI and 51°C for TcII both at 30 s, extension at 72°C for 30 s, and a final extension at 72°C for 10 min. Amplicons were visualized as described in the previous section. To confirm Tc types, a phylogenetic tree was constructed with the sequences of amplicons and their best hits with GenBank (BLASTN search) as described in the previous section.

### PCR amplification of the 16S rRNA gene fragments for DGGE analysis

To evaluate the correlation between the presence of *T. cruzi* infection and the composition of bacterial gut microbiota, we amplified the 16S rRNA gene fragments of bacterial communities corresponding to the V6-V8 region of the *E. coli* 16S rRNA. The same approach was also used to evaluate changes in the bacterial gut microbiota of *Triatoma* specimens kept in the insectary over time. All metagenomic DNA samples were amplified using the primer pair F968-GC (5′ – CGC CCG CCG CGC CCC GCG CCC GTC CCG CCG CCC CCG CCC G AAC GCG AAG AAC CTT AC-3′) and L1401 (5′ CGG TGT GTA CAA GAC CC- 3′) described by Nübel et al. [36] using a thermocycler (Applied Biosystems, CA, USA).

PCR amplifications were carried out containing approximately 50 ng of metagenomic DNA template, 1X reaction buffer, 0.25 mM MgCl_2_, 0.2 μM dNTP, 0.2 μM of each primer, 0.125 U GoTaq DNA polymerase (Promega, USA) and water to a final volume of 50 μl. All amplification reactions started at 94°C for 2 min followed by 35 cycles of denaturation at 94°C for 1 min, annealing at 48°C for 1.5 min, and extension at 72°C for 1.5 min with a final extension at 72°C for 10 min. Amplicons were analyzed as described in the previous section and stored at −20°C until DGGE analysis.

### DGGE of 16S rRNA gene fragments and band sequencing

The microbiota fingerprints of all gut samples in this study were obtained using DGGE as described in da Mota et al. [26] with the D-code system (Bio-Rad Laboratories, Munich, Germany). Amplicons of bacterial 16S rDNA were loaded onto a 6% polyacrylamide gel that was prepared with a denaturing gradient in the range of 45% - 65%. The denaturing agent (100%) was a solution of urea (7 M) and formamide (40%). Electrophoresis was run in TAE (Tris acetic EDTA) buffer at 60°C for 16 h at 75 V. After electrophoresis, gels were stained with SYBR Green I (Sigma Aldrich, MO, USA) and subsequently digitized using a Thyphoon Trio scanner (GE Healthcare Life Science, USA) with 400 nm excitation and 520 BP emission filters. Dominant bands (thicker bands) were excised from DGGE gels and transferred to 1.5 ml tubes. The gel slices were crushed with the top of a sterile tip and eluted in 50 μl of water for sequencing. The eluent was used as PCR template DNA using primers U968 and L1401. DNA sequencing was performed with an ABI Genetic Analyzer (Applied Biosystems, CA, USA). The partial 16S rDNA sequences from DGGE bands were assigned to uncover the taxa positions of bacteria until the genus level using the SeqMatch program [37] of the Ribosomal Database Project (RDP) to preliminarily identify bacterial microbiota members.

### PCR conditions for full-length 16S rRNA cloning and Sanger sequencing

To more precisely identify the taxonomical position of bacteria from DNA samples of *T. brasiliensis* (35 Tb and 37 Tb) and *T. pseudomaculata* (17Tp) guts, which showed high species diversity, we opted for full-length sequencing of 16S rDNA. The amplification of full-length 16S rDNA was carried out with forward primer pA (5′-AGA GTT TGA TCC TGG CTC AG-3′) and reverse primer pH (5′-AAG GAG GTG ATC CAG CCG CA-3′), according to Massol Deya [38]. PCR conditions were performed as described in da Mota et al. [26]. Amplicons with the expected molecular length were excised from the agarose gel (1% w/v) after electrophoresis and purified with a QIAquick Gel Extraction Kit (Qiagen, Hilden, Germany). Purified amplicons were ligated into the pGEM-T Easy plasmid vector (Promega, Madison, WI, USA) and sequenced by the Sanger method with the Big Dye reagent version 3.1 (Applied Biosystems, Foster City CA, USA) with forward and reverse M13 primers (designed for sequencing inserts in the plasmid vector pGEM-T) and a universal primer 341f (5′- CCT ACG GCA GGC AGC AG -3′) using an Applied Biosystems ABI Prism 3730 xl automated DNA sequencer to obtain the full length of the 16S rDNA inserts. The completed sequences (21) were deposited in GenBank under the accession numbers KP713412 - KP713432. To better identify the bacterial species, we constructed a phylogenetic tree as described before, with the amplicon sequences and the sequences of their best hits with GenBank [31].

### Amplification and 454 pyrosequencing of the variable region of the 16S rRNA gene

For quantitative analysis of the microbiota, 16S rDNA amplicons from gut samples of two specimens of *T. brasiliensis* (35 Tb and 37 Tb) and two specimens of *T. pseudomaculata* (17Tp and 19Tp) were submitted to FLX-Titanium for pyrosequencing. Samples were amplified and sequenced using barcoded primers for the 16S variable region V3-V1 according to the HMP 3730 16S Protocol version 4.2 [27] available from the HMP Data Analysis and Coordination Center website [39].

A total of 76,842 raw sequences from the four *Triatoma* samples were processed with the GL FLX proprietary software. Sequences (44,262) with a score below the FLX quality threshold were discarded and the sequence portions devoted to 454 sequencing were trimmed out. Sequences were then aligned using the INFERNAL aligner [40]. Chimeric sequences were detected and removed with UCHIME [41]. Cleaned sequences were analyzed using the RDP Pipeline [42] and taxonomical classification was assigned using the RDP classifier [37] with a minimum confidence level for record assignment set to 0.90. To estimate the diversity, we used the Good’s coverage estimator (calculated as [1-(n/N)] x 100, where n is the number of singleton phylotypes and N is the number of sequences), Shannon diversity index, and Chao1 estimation of species richness. The Shannon diversity index and the Shannon equitability index were calculated using the equation H = −Ʃ*RA*_i_ln(*RA*_i_), and *E*_*H*_ = *H*/ln(*S*), respectively (where *RA*_i_ is the proportion of the *i*^th^ OTU and *S* is the total number of OTUs) [43].

## Results

### Morphological and molecular characterization of triatomines collected in peridomestic habitats

All 33 triatomine specimens (males and female adults and nymphs of the 3^rd^, 4^th^ and 5^th^ stage) were identified as belonging to the *Triatoma* genus using morphological features (Table 1). *Triatoma* species with similar morphological features are endemic in Russas, which motivated the molecular approach based on COI sequencing (barcoding) for unequivocal species identification. COI confirmed the morphological identification of 13 *T. pseudomaculata* and 20 *T. brasiliensis* specimens (Table 1)*.*

**Table 1:** **DGGE profile variation of** ***Triatoma*** **sp.,** ***Trypanosoma cruzi*** **detection and developmental stage**

**Sample**	***Triatoma*** **species**	**Infection**		**DGGE profile**	**Developmental stage**
		**TcI**	**TcII**		
9Tp	*T. pseudomaculata*	P	P	A	Ad♂
10Tp	*T. pseudomaculata*	N	P	C	Ad♀
12Tp	*T. pseudomaculata*	N	P	A	Ad♂
13Tp	*T. pseudomaculata*	N	P	D	Ad♀
14Tp	*T. pseudomaculata*	N	P	F	Ad♂
15Tp	*T. pseudomaculata*	N	P	E	Ad♀
16Tp	*T. pseudomaculata*	N	P	B	Ad♀
17Tp	*T. pseudomaculata*	N	P	G	N5
19Tp	*T. pseudomaculata*	P	P	H	Ad♂
20Tp	*T. pseudomaculata*	N	N	I	Ad♀
21Tp	*T. pseudomaculata*	N	N	J	Ad♀
22Tp	*T. pseudomaculata*	ND	ND	N	Ad♀
50Tp	*T. pseudomaculata*	N	N	N	N5
24Tb	*T. brasiliensis*	N	N	C	Ad♂
25Tb	*T. brasiliensis*	P	P	K	Ad♀
26Tb	*T. brasiliensis*	P	P	C	Ad♀
27Tb	*T. brasiliensis*	P	P	L	Ad♀
28Tb	*T. brasiliensis*	P	N	C	Ad♂
29Tb	*T. brasiliensis*	P	P	C	Ad♂
30Tb	*T. brasiliensis*	P	P	C	Ad♂
31Tb	*T. brasiliensis*	P	P	C	Ad♂
32Tb	*T. brasiliensis*	N	P	A	Ad♂
33Tb	*T. brasiliensis*	P	P	C	Ad♂
35Tb	*T. brasiliensis*	P	P	M	N5
37Tb	*T. brasiliensis*	P	P	C	N5
38Tb	*T. brasiliensis*	P	N	C	N5
39Tb	*T. brasiliensis*	P	N	C	N5
40Tb	*T. brasiliensis*	P	N	C	N5
41Tb	*T. brasiliensis*	P	N	E	N4
42Tb	*T. brasiliensis*	N	N	C	N4
43Tb	*T. brasiliensis*	P	N	C	N3
48Tb	*T. brasiliensis*	P	N	A	N5
49Tb	*T. brasiliensis*	P	N	A	N5

All triatomines were collected in Russas - Ceará, Brazil. The taxonomical position of all triatomines was confirmed with the mitochondrial COI gene marker. The DGGE profile was identified using the 16S rRNA gene fragments (V6-V8). ♀ or ♂ stand for female or male adults (Ad), respectively. N3, N4 and N5 stand for nymph at 3^rd^, 4^th^ and 5^th^ stage, respectively. P and N stand for positive and negative PCR amplification while ND stand for undetermined.

**Figure 1:**
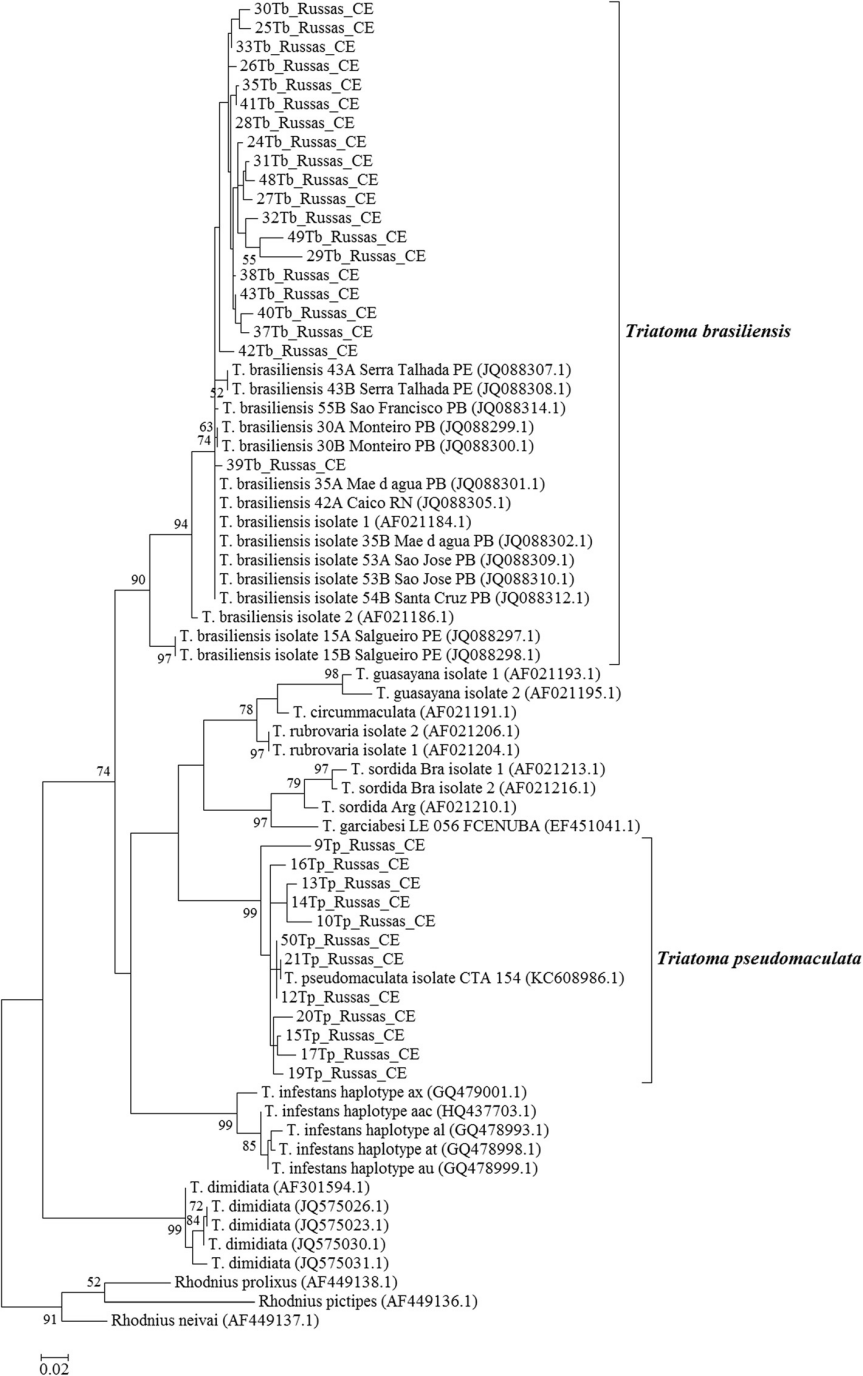
Maximum likelihood phylogenetic tree from the mitochondrial COI region of peridomestic triatomine species*.* The 32 sequences were obtained from specimens of *T. brasiliensis* and *T. pseudomaculata* collected in peridomestic habitats (Russas, Ceará, Brazil). They were compared with other sequences of *Triatoma* sp. obtained from GenBank. *Rhodnius* spp*.* were used to form an outgroup. The relative values (%) on branches are based on 1,000 bootstrap replicates.

Therefore, 32 COI gene fragments were sequenced and aligned with the top ten sequences available from GenBank for *Triatoma* species (*T. brasiliensis*, *T. guasayana*, *T. circunmaculata*, *T. rubrovaria*, *T. sordida*, *T. garciabesi*, *T. pseudomaculata*, *T. infestans*, *T. dimidiata*) and *Rhodnius* species (*R. neivai*, *R. pictipes*, *R. prolixus*), used as an outgroup (Figure 1). By reference to the phylogenetic tree derived from the COI sequence alignment, all sequences obtained from insects captured in Russas grouped into two clades of *Triatoma*. The first clade, supported by a bootstrap value of 90%, included 20 specimens from Russas closely related to the sequences of *T. brasiliensis* from other cities of the northeast region in Brazil. The other clade, supported by a bootstrap value as high as 99%, included 12 specimens from Russas closely related to the sequences of *T. pseudomaculata,* also found in Brazil and belonging to the *T. pseudomaculata* subcomplex.

### Distribution of *Trypanosoma cruzi* types (TcI and TcII) in *Triatoma* specimens collected in peridomestic habitats

**Figure 2:**
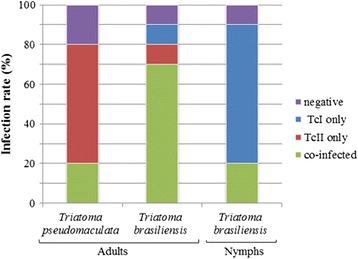
Percentage of *T. cruzi* (TcI and TcII) from *T. pseudomaculata* and *T. brasiliensis.* Adults and nymphs were collected in the peridomestic habitats of Russas (Ceará, Brazil). *T. cruzi* strains were genotyped by the *mini-exon* gene marker.

A total of 32 triatomine specimens (12 for *T. pseudomaculata* and 20 for *T. brasiliensis*) were examined through the mini-exon gene for assessment of *T. cruzi* infection. The overall rate of infection by *T. cruzi* in *T. pseudomaculata* and *T. brasiliensis* was 80% and 90%, respectively (Figure 2). In *T. pseudomaculata,* 60% of individuals were positive for TcII only and 20% showed mixed co-infection by TcI and TcII (Figure 2). However, in *T. brasiliensis* 10% of adult triatomines showed infection for TcI only and 10% for TcII only, while 70% had mixed co-infections with TcI and TcII (Figure 2). By contrast, in *T. brasiliensis* nymphs, infection occurred mainly with TcI only (70%), while co-infection by TcI and TcII occurred in 20% of cases (Figure 2). PCR-negative for TcI and TcII ranged from 10 to 20% of specimens in all groups analyzed (Figure 2).

### Evaluation of Tc type specificity by PCR in metagenomic DNA

**Figure 3:**
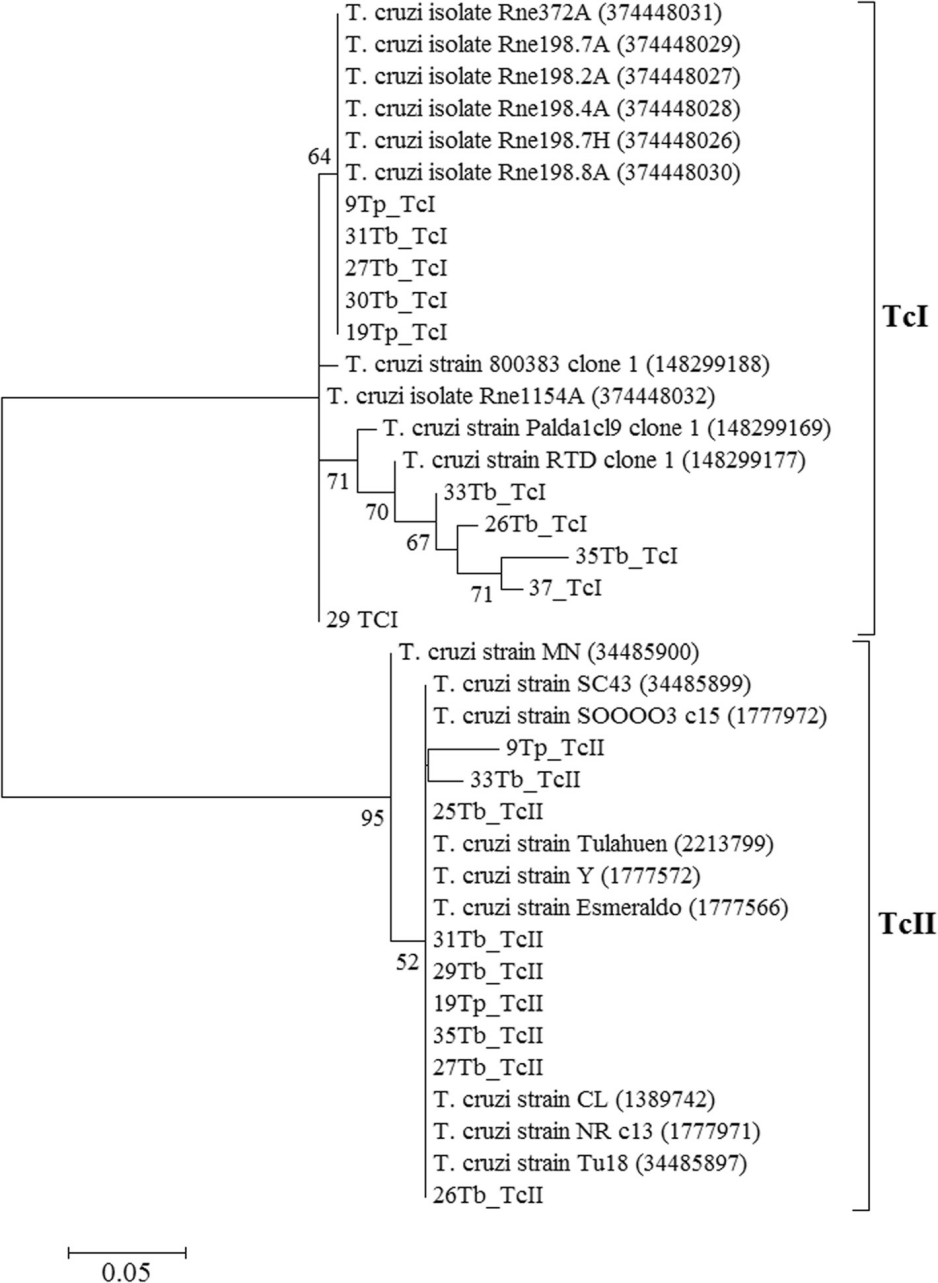
Maximum likelihood phylogenetic tree of the *mini-exon* gene from *T. cruzi.* Representative sequences identified as TcI and TcII strains from *T. brasiliensis* and *T. pseudomaculata* collected in peridomestic habitats (Russas, Ceará, Brazil) were compared with other sequences of *Triatoma* sp. obtained from GenBank. The relative values (%) on branches are based on 1,000 bootstrap replicates.

To confirm the specificity of singleplex PCR conditions for the diagnosis of Tc types in metagenomic DNA, partial sequencing (200–250 bp) of some *T. cruzi* mini-exon genes was carried out by the Sanger method. Nineteen sequences of *T. cruzi* mini-exon gene from *T. brasiliensis* and *T. pseudomaculata* infected with *T. cruzi* were aligned with the sequences of reference strains of TcI and TcII deposited in GenBank. The phylogenetic tree obtained showed two major clades within *T. cruzi* corresponding to TcI and TcII (Figure 3). The sequences amplified with TcI primers were grouped into the TcI clade together with the reference sequences of this clade, while another set of sequences amplified with TcII primers grouped into a clade derived from TcII reference sequences. *T. cruzi* types differing from TcI and TcII were detected by similarity comparison with GenBank in some co-infected samples (Figure 3).

### DGGE profiles of bacterial microbiota in *Triatoma* reared in the insectary over different periods of time

**Figure 4:**
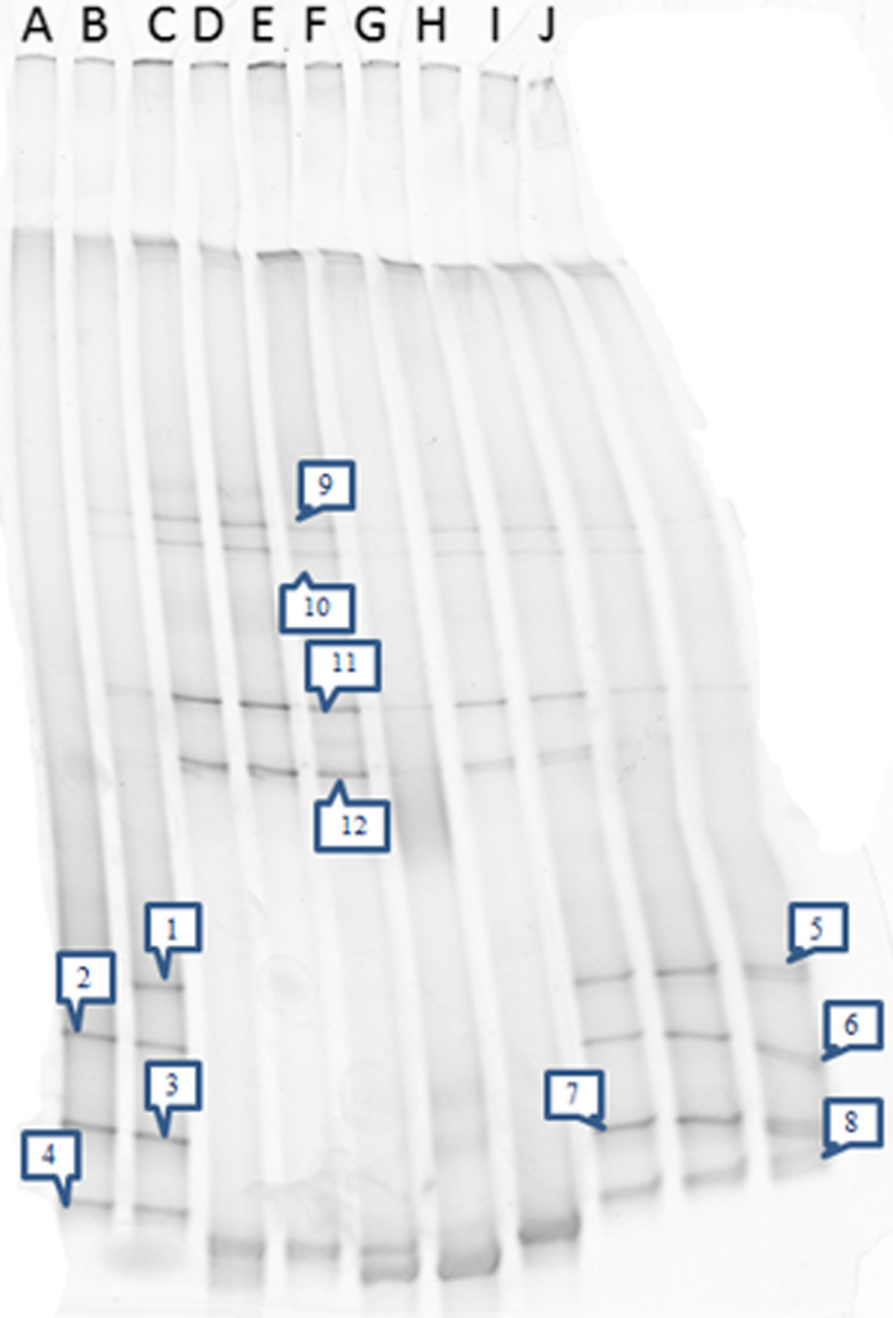
DGGE fingerprints of bacterial 16S rRNA gene fragments amplified from gut microbiota. Lanes correspond to **(A)** *T. infestans* and **(B)** *T. vitticeps* specimens reared in the insectary (Fiocruz/IOC) from the five year colony, **(C to G)** *T. brasiliensis* from the few weeks colony, and **(H-J)** *T. brasiliensis* from the three year colony. Bands 1 to 8 and 9 to 12 correspond to *Arsenophonus* and *Serratia* genera, respectively.

The DGGE profiles of *T. brasiliensis* maintained or reared for a few weeks (lanes C to G), three years (H to J) and five years (lanes A and B) differed in the composition of gut microbiota (Figure 4). DGGE profiles were the same for each replicate of the same category. Band sequencing revealed that the bacterial genera corresponding to bands 1 to 8 were essentially members of *Arsenophonus.* Those corresponding to bands 9 to 12 essentially belonged to *Serratia* (Figure 4). Bacterial composition changes were observed when triatomines passed from field to laboratory conditions, with a reduction in the *Serratia* population balanced by an increased contribution of the intracellular *Arsenophonus*.

### DGGE profiles of the bacterial microbiota in *Triatoma* collected in peridomestic habitats

**Figure 5:**
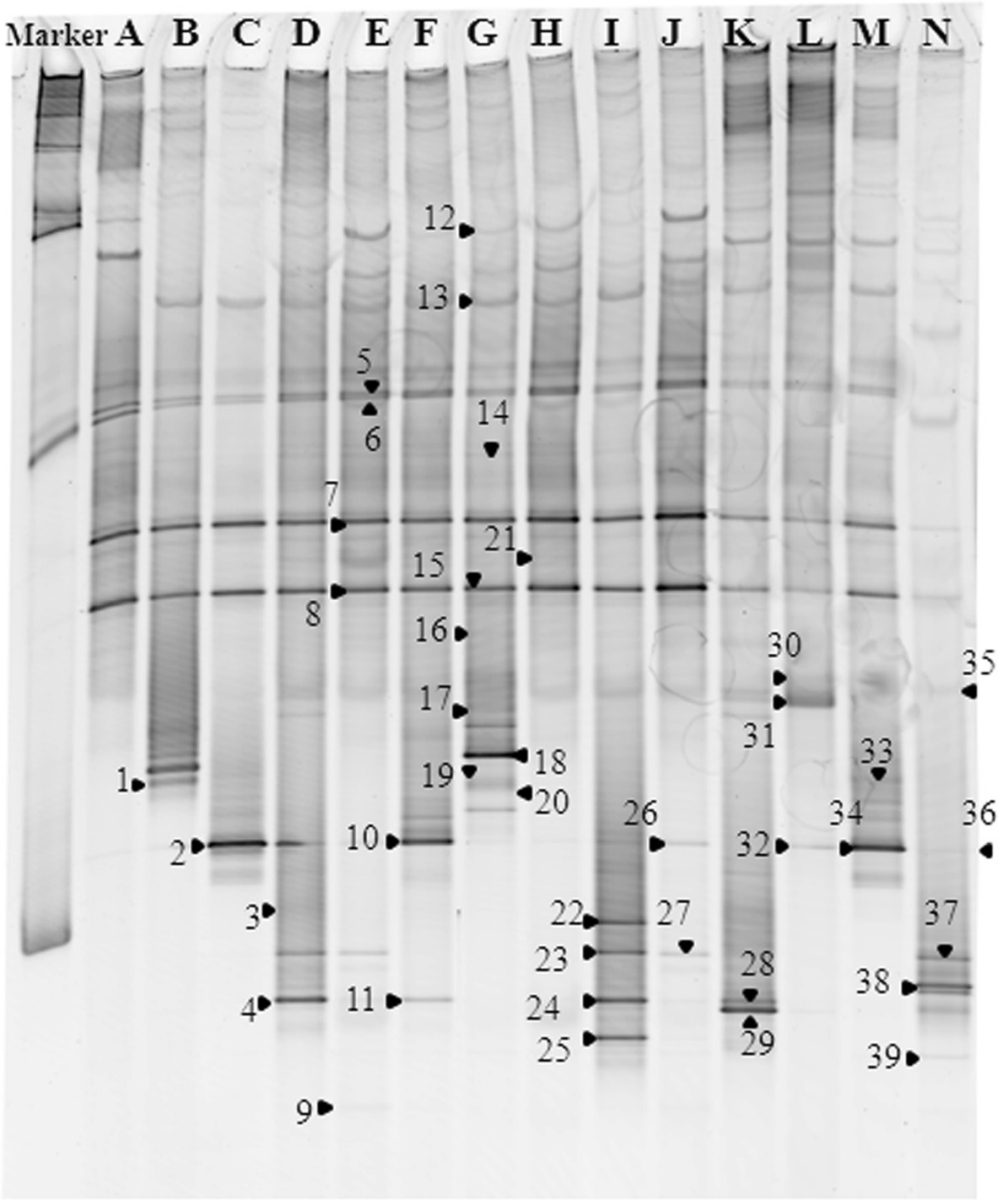
DGGE fingerprints of bacterial 16S rRNA gene fragments amplified from gut microbiota of *T. pseudomaculata* (Tp) and *T. brasiliensis* (Tb) collected in peridomestic habitats (Russas, Ceará, Brazil). A - N correspond to the 14 band profiles identified in the triatomine samples 32Tb, 16Tp, 37Tb, 13Tp, 15Tp, 14Tp, 17Tp, 19Tp, 20Tp, 21Tp, 25Tb, 27Tb, 35Tb, and 50Tp, respectively. Numbers indicate sequenced bands: *Serratia* (5 to 8, 12, 13 and 15); *Pantoea* (31); *Enterococcus* (1 and 19); *Bacteroidetes* (35); *Gordonia* (2, 10, 26, 32 and 34); *Mycobacterium* (3, 4, 9, 11, 22 to 25, 27, 29 and 39); *Corynebacterium* (20); *Dietzia* (18); *Rhodococcus* (28, 37 and 38); Enterobacteriaceae (14, 16, 17, 20, 21 and 30); and Nocardiaceae (33 and 36).

DGGE fingerprints of bacterial communities from 13 *T. pseudomaculata* gut samples showed 11 different DGGE profiles (A, B-J and N), whereas only two specimens presented the same profile (A and N) (Figure 5 and Table 1). By contrast, in *T. brasiliensis* we observed only six (C, A, E, K, L and M) different DGGE profiles from 20 gut samples, with 13 samples belonging to the C profile and three to the A profile (Figure 5 and Table 1).

The SeqMatch analysis of sequences associated with the most representative DGGE bands showed that the bacterial microbiota belonged to nine different genera corresponding to four phyla: Proteobacteria, Actinobacteria, Firmicutes and Bacteroidetes (Figure 5). The sequences associated with DGGE bands allowed the diagnosis of the following genera by reference to their closest matches in SeqMatch, with at least 99% sequence identity: *Serratia* (bands 5 to 8, 12, 13 and 15); *Pantoea* (band 31); *Enterococcus* (bands 1 and 19); *Bacteroidetes* (band 35); *Gordonia* (bands 2, 10, 26, 32, and 34); *Mycobacterium* (bands 3, 4, 9, 11, 22 to 25, 27, 29 and 39); *Corynebacterium* (band 20); *Dietzia* (band 18); *Rhodococcus* (bands 28, 37 and 38); Enterobacteriaceae (bands 14, 16, 17, 20, 21 and 30); and Nocardiaceae (bands 33 and 36) (Figure 5). *Serratia*, *Gordonia* and *Mycobacterium* were the predominant genera in peridomestic specimens of *Triatoma* sp. (Figure 5).

### Phylogeny and identification of bacterial species in the gut microbiota of *Triatoma* collected in peridomestic habitats using a clone library of the 16S rRNA gene

**Figure 6:**
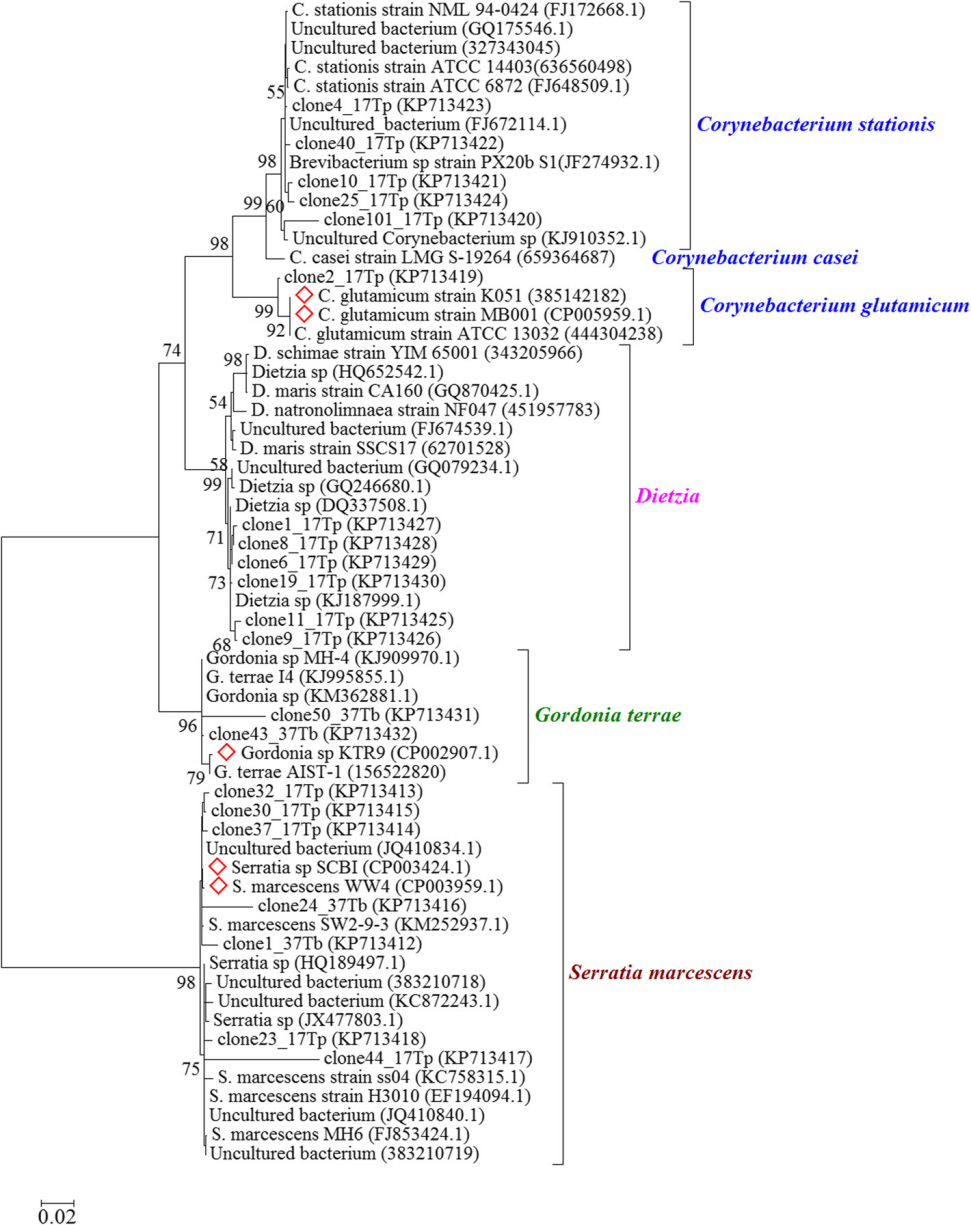
Maximum likelihood phylogenetic tree of 16S rRNA gene of microbiota in triatomine guts*.* The 21 representative partial sequences identified as bacterial genes from gut microbiota of *T. pseudomaculata* (Tp) and *T. brasiliensis* (Tb) collected in peridomestic habitats (Russas, Ceará, Brazil) were compared with other partial sequences obtained from GenBank. Red diamonds represent sequences obtained from species with complete genomes. The percentages on branches are based on 1,000 bootstrap replicates.

A phylogenetic tree constructed from 21 sequences of the complete 16S rRNA gene cloned from gut microbiota and their 10 best hits with sequences in GenBank displayed four major branches related to *Corynebacterium* spp., *Gordonia* spp., *Dietzia* spp. and *Serratia* spp. (Figure 6). Some clones from *T. pseudomaculata* (17Tp) were grouped close to *Corynebacterium stationis*, while another clone was grouped close to *Corynebacterium glutamicum*. Clones related to *Dietzia* sp. were also found, but the species identification was not possible using a diagnosis based on the sequences of 16S rDNA amplicons alone. Clones related to *Gordonia terrae* clustered close to *Gordonia* sp*.* The KTR9 genome with clones related to *Serratia marcescens* clustered in two branches corresponding to (i) WW4 and SCBI strains whose genomes were sequenced, and (ii) MH6, H3010 and SS04 strains. Both branches include uncultured *Serratia* sequences (JQ410834 and JQ410840) previously isolated from the *R. prolixus* gut. Some clinical isolates of *C. stationis* (NML 94–0424 and ATCC 14403) and *Dietzia maris* (CA160) clustered close to the 16S rDNA sequences of gut samples from *T. pseudomaculata* captured in peridomestic habitats.

### Pyrosequencing of the 16S rRNA gene and quantitative analysis of bacterial gut microbiota in *Triatoma* collected in peridomestic habitats

By 454 pyrosequencing of 16S rDNA from gut samples of four different triatomines, 32,580 sequences were obtained and classified into 30 bacterial genera (Table 2). The most abundant bacterial genera found in *T. brasiliensis* were (i) *Mycobacterium* sp. (74%), *Rhodococcus* sp. (24%) and *Serratia* sp. (1%) in 25 Tb and (ii) *Gordonia* sp*.* (96%) and *Serratia* sp. (3%) in 35 Tb (Table 2). In *T. pseudomaculata*, the most abundant bacterial genera were (i) *Dietzia* sp. (67%), *Corynebacterium* sp*.* (27%) and *Serratia* sp. (5%) in 17Tp and (ii) *Serratia* sp. (97%) followed by other genera such as *Mycobacterium* sp., *Hydrogenophilus* sp., *Williamsia* sp. and *Gordonia* sp., each accounting for less than 1%, in 19Tp (Table 2). Although differences in bacterial members were observed for each individual, the gut microbiota of *Triatoma* was composed predominantly by genera from the Corynebacterineae suborder (81%), such as *Corynebacterium*, *Dietzia*, *Gordonia*, *Mycobacterium*, *Rhodococcus* and *Williamsia* (bolded in Table 2). *Serratia*, a member of Enterobacteriaceae, was the only genus encountered in all samples analyzed; it represented 18% of all sequences obtained by 454 pyrosequencing.

**Table 2:** **Identification of bacterial genera by pyrosequencing of 16S rDNA from the microbiota of triatomine guts**

**Bacterial genus**	**25TB**	**35TB**	**17Tp**	**19Tp**
*Acinetobacter*		1		2
*Actinomyces*			1	
*Adhaeribacter*				1
*Bradyrhizobium*	1			
*Chryseobacterium*				1
*Comamonas*		1		
Corynebacterium*		**1**	**2015**	
*Diaphorobacter*			2	1
Dietzia*		**1**	**5008**	
*Enterococcus*			2	6
*Geobacillus*		2		
Gordonia*	**1**	**11825**		**10**
*Haemophilus*		1		
*Hydrogenophilus*		6	1	15
*Janthinobacerium*		1		
*Marinomonas*				1
*Microvirga*				2
Mycobacterium*	**5737**			**27**
*Propionibacterium*		1		5
*Pseudomonas*		4	3	2
Rhodococcus*	**1855**			**5**
*Serratia*	116	426	422	4917
*Shinella*				2
*Sphingomonas*				1
*Staphylococcus*	1	3		
*Stenotrophomonas*			1	
*Streptococcus*		1		1
*Streptphyta*		2		1
Williamsia*		**3**		**15**
*Xanthobacter*		1		
Not assigned (genus > 90% bootstrap)	52	13	9	45

Triatomines corresponding to *Triatoma brasiliensis* (25Tb and 35Tb) and *T. pseudomaculata* (17Tp and 19Tp) were collected in peridomestic habitats from Russas (Ceará, Brazil). The numbers in the table indicate the absolute number of times each genus was detected in each sample. Bold names with an asterisk indicate bacterial genera belonging to the Corynebacterineae suborder.

**Figure 7:**
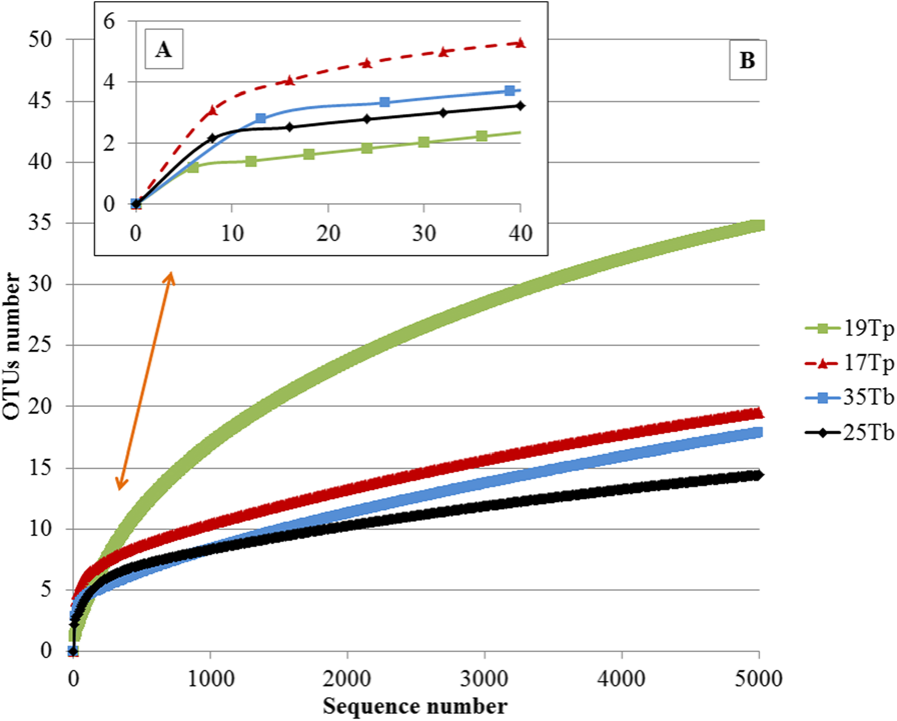
Rarefaction curves of 16S rDNA sequences from triatomine gut microbiota. The rarefaction curves were calculated using the RDP pipeline in libraries of *T. brasiliensis* (25Tb and 35Tb) and *T. pseudomaculata* (17Tp and 19Tp) 16S rDNA fragments. Panel **A**: The number of different bacterial species is given as a function of the number of sequences obtained by pyrosequencing. Panel **B**: In contrast to *T. pseudomaculata* (19Tp) that does not reach species saturation even with the sequencing of 5,000 sequences, the microbiota of *T. brasiliensis* (25Tb and 35Tb) and *T. pseudomaculata* (17Tp) approached saturation with less than 100 sequences. The OTU numbers are referred to 0.05.

Rarefaction curves (Figure 7A and B) obtained for 25 Tb, 35 Tb and 17Tp show that the microbiota of these triatomine samples was pyrosequenced close to saturation. By contrast, the rarefaction curve of 19Tp showed that the microbiota of this sample was not fully covered (Figure 7B). Therefore, the real spectrum of bacterial species for this sample could indeed be larger than the 19 bacterial genera reported here (Table 2; Figure 7A). However, deeper sequencing would not affect the general trend outlined. The values of the Good’s coverage estimator calculated from the 16S rDNA sequences of *T. brasiliensis* (25Tp and 35Tp) and *T. pseudomaculata* (17Tp and 19Tp) libraries ranged from 99.3 to 99.8, confirming the representativeness of our results compared to the real condition (Additional file 1). The Shannon-Wiener diversity index (H’) value was the largest in 17Tp and influenced by the largest equitability among the 23 OTUs (clusters). On the other hand, 19Tp showed the lowest equitability (Additional file 1) due to the *Serratia* dominance (Table 2) but the largest number of clusters with 35 OTUs (Figure 7A, B). It also exhibited the largest number of genus affiliations with 19 genera (Table 2). However, the relative importance of these genera is marginal relative to *Serratia,* as proven by the low sample equitability and, as shown by the rarefaction curve of Figure 7B, is the product of the deep coverage attained by 454 pyrosequencing.

## Discussion

The insect gut is often colonized by multispecies microbial communities that play integral roles in insect physiology and defense against parasite infections. Therefore, a description of the interactions between *T. cruzi*, triatomines and the bacterial microbiota is important to understand the insect vector competence for Chagas disease transmission. With this concern, we analyzed the gut bacterial microbiota as well as TcI and TcII types of *T. cruzi* in *T. brasiliensis* and *T. pseudomaculata* collected from peridomestic ecotopes in the northeastern region of Brazil and characterized them by barcoding in reference to COI.

The triatomine species *T. pseudomaculata* and *T. brasiliensis* are endemic in the state of Ceará (Brazil) and were easily found in 13 municipalities where Chagas disease is reported [44]. Because *T. brasiliensis* naturally occupies domestic, peridomestic and sylvatic environments, the probability of interaction between triatomine and humans is high [7,45]. Therefore, particular attention is required by health authorities in localities where high indices of triatomine species re-infestation have been identified [46]. Unlike the successful eradication of domestic *T. infestans* by insecticide treatments, the control of peridomestic species such as *T. brasiliensis* and *T. pseudomaculata* proved to be ineffective [47,48] because of their natural habitat acting as a reservoir for these bugs.

COI is a good marker for population studies due to its consistent polymorphisms in triatomines and the accurate phylogenetic information it provides [49]. The construction of a phylogenetic tree from mitochondrial COI sequences obtained from the triatomines of this study allowed their taxonomical classification at nymphal stages and confirmation based on morphological characteristics at the adult stage. The *T. brasiliensis* specimens grouped into a monophyletic branch exclusively composed of *T. brasiliensis* within the clade that included reference sequences from GenBank for this species, all from the northeastern region of Brazil [50,51]. With regard to *T. pseudomaculata,* our sequences are closely related to the sequences of triatomine specimens also found in Brazil and belonging to *T. pseudomaculata* [49,50,52].

*T. brasiliensis* and *T. pseudomaculata* are synanthropic species in the northeastern region with high rates of natural infection by *T. cruzi* [3,53]. Using specific primers for the mini-exon region, we detected 90% and 80% infection rates by *T. cruzi* (TcI and TcII) in *T. brasiliensis* and *T. pseudomaculata*, respectively.

The type of *T. cruzi* that can be isolated from triatomine guts is known to vary according to the specific physiology and ecological habit of these vector bugs [13,54-57]. The genetic complexity of *T. cruzi* may explain the different TcI and TcII proportions in nymphs and adults of *T. pseudomaculata* and *T. brasiliensis* from Russas. More precisely, the measurement by PCR of the infection rate due to *T. cruzi* in triatomines will depend on the molecular markers used for its diagnosis and on the type of strains present at the moment of the measurement. For instance, markers such as 24α rRNA, 18S rRNA or others may detect a larger range of *T. cruzi* types. The primers we used were designed by Fernandes et al. [11]. These primers have a resolution sufficient for a gross diagnosis of *T. cruzi* presence or absence, but not for its precise taxonomic classification in six types (TcI – TcVI) [13]. However, *T. cruzi* classification depends on markers that are effectively used and do not necessarily reflect the infective or ecological peculiarities of the corresponding strains. The PCR we used to diagnose *T. cruzi* according to Fernandes et al. [11] showed that the triatomine rate of infection by this parasite was much larger than expected, which raises concerns for the epidemiology of Chagas disease in Russas. Differences in infection rates between nymphs and adults of both *Triatoma* species could be related to features such as flight mobility (only available at adult stage), coprophagy, species of animals used as feeding sources or numbers of feeding individuals (adults usually had one or more blood ingestion than nymphs). In addition to the fact that both triatomine vectors circulate in peridomestic and domestic areas, *T. brasiliensis* is found in a larger ecotope variety and may deserve more attention than *T. pseudomaculata* because it is able to transmit *T. cruzi* [8,58-60].

In triatomines from the insectary, the composition of the gut bacterial microbiota is selective to some degree of each triatomine genus [26]. *T. infestans* and *T. vitticeps* presented the largest diversity of gut bacteria compared to *R. prolixus*, *Panstrongylus megistus* and *Dipetalogaster maximus* [26].

The diversity of the gut microbiota of *T. brasiliensis* and *T. pseudomaculata* collected in peridomestic ecotopes analyzed by DGGE of the 16S rRNA gene fragments (V6-V8 region) showed a relatively low average of bands per sample. We found 14 different DGGE profiles among the 33 specimens analyzed. Interestingly, we could not find any alteration of DGGE profiles that could be correlated to triatomine infection by *T. cruzi* in field conditions. The low level of species diversity of bacterial communities from triatomine guts was confirmed by pyrosequencing. As expected, more OTUs were identified with respect to those found through DGGE analysis and sequencing of full length 16S rDNA. The low level of microbiota diversity has generally been observed in hematophagous insects [61,62], but contrasts with that reported for other non-hematophagous insect taxa such as termites, which house the richest microbial communities found in insects [63] due to a complex coevolution between the host insect and its microbiota composition [61,64]. The restricted blood feeding lifestyle of triatomines is supposed to contribute to the low microbiota diversity detected in the gut of field collected nymphs and adults of *T. brasiliensis* and *T. pseudomaculata*, regardless of the investigation methods. In addition, the blood substrate itself is sterile, which diminish the chance of contamination with complex microbiota by triatomines. However, selection pressures were also proposed to shape the gut bacterial community in triatomines through immune responses depending on starvation and feeding conditions [65].

We found that members of the Actinobacteria phylum were quantitatively and qualitatively variable in gut microbiota and presented different genera and species according to the case considered. Actinobacteria exhibit diverse physiological and metabolic properties, such as the production of extracellular enzymes and the formation of a wide variety of secondary metabolites and antimicrobial bioactive compounds [66]. We observed that the gut microbiota of *T. brasiliensis* and *T. pseudomaculata* are overwhelmingly dominated by Actinobacteria belonging to the suborder Corynebacterineae (Actinomycetales). The Corynebacterineae, is composed by antibiotic producing bacteria such as *Dietzia*, *Corynebacterium*, *Rhodococcus*, *Mycobacterium* and *Gordonia* that are widely found in acid soils [66-70]. Once triatomines have coprophagic habits, Corynebacterineae might possibly be acquired through the ingestion of feces contaminated with soil, which might explain their varying populations in the gut of triatomines from the field [71]. Interestingly, some 16S rDNA sequences from *Triatoma* gut samples are closely related to clinical isolates belonging to *C. stationis* and *Gordonia terrae* obtained from blood cultures [72,73], supporting the idea that these bacteria have an enzymatic system enabling the use of blood as a nutrient source. *R. rhodnii*, a symbiont of *R. prolixus* that has been reported to provide B complex vitamins [74,75], was also found in some specimens of *T. pseudomaculata* and *T. brasiliensis*. On the other hand, we generally found species of the *Corynebacterium* genus, which was described as a symbiont of *T. infestans* [76]. This genus participates in the supply of pantothenic acid [76].

The phylum Proteobacteria was represented by *S. marcescens* found in all analyzed specimens from field and insectary, suggesting that this species is well adapted to this environment and is likely transmitted from insect to insect or even vertically. Moreover, the co-existence of *S. marcescens* and many Corynebacterineae in the triatomine gut environment suggests that *S. marcescens* has resistance genes against antibiotics produced by Corynebacterineae [77]. Proteobacteria were major members of the bacterial communities found in all triatomine gut samples analyzed and represent the predominant bacterial phylum in the gut microbiota of various invertebrates such as the ground beetle, the *Lutzomyia* sand fly, *R. prolixus* and the desert locust *Schistocerca gregaria* [17,78-80]. Members of the family Enterobacteriaceae within Proteobacteria constitute the majority of the gut microbiota in insects, as is also the case for fruit fly species [81,82]. The wide distribution of Enterobacteriaceae suggests important functions for insect hosts [83,84]. Gut Enterobacteriaceae communities may indirectly contribute to host fitness by competing with pathogenic microorganisms [16,17,19,61,85].

*Serratia* was the most represented Enterobacteria in guts of *T. brasiliensis* and *T. pseudomaculata* from the field as well as in triatomines reared in the insectary. Although we found *Serratia* in the guts of all triatomine specimens through three different techniques, we could not find a clear relationship between *Serratia* and the presence of *T. cruzi* (TcI or TcII). The facultative anaerobe *S. marcescens* biotype A1 isolated from the gut of *R. prolixus* has been reported to have trypanolitic activity, especially on the Y strain [85]. Prodigiosin, a pigment in some strains of these bacteria, has been proposed to be responsible for trypanocidal activity [85,86]. However, Castro et al. [87,88] proposed that mannose sensitive fimbriae might be directly involved in the trypanocidal activity by connecting bacteria to the surface of the parasite and promoting their adhesion to the protozoan cellular membrane.

Although trypanocidal activity by *Serratia marcescens* was reported in *in vitro* conditions [85], neither *Serratia*, *Dietzia*, *Gordonia*, *Mycobacterium*, *Corynebacterium* nor *Rhodococcus* ensure protection to prevent triatomines from infection by *T. cruzi* alone or in combination.

Another possibility is that bacteria might play specialized functions according to the distinct regions of the gut. Here, we amplified bacterial 16S rDNA from the whole gut. Thus, any correlation between distinct gut regions that bacteria may preferentially colonize cannot be shown [89].

Enterobacteria of the genus *Arsenophonus* were found in insects reared in the insectary, but not in specimens of *T. brasiliensis* and *T. pseudomaculata* collected in the field. As suggested above, gut bacterial diversity in natural conditions could be of higher complexity due to a larger likelihood of contact with soil bacteria. By contrast, the transmission of some species of bacteria, such as *Arsenophonus*, could be facilitated by the insectary life style. The uniform insectary conditions for temperature, humidity, feeding sources, host feeding state high insect density and absence of soil could favor some species of bacteria and lead to the modification of microbiota composition of triatomine guts over time [90,91]. In the present investigation, the bacterial gut microbiota of insect colonies differed from the wild according to how long the colony is maintained in the insectary.

Any techniques present specific benefits and drawbacks. Previous investigations based on the culture of bacteria on artificial media showed the predominance of Proteobacteria and of Actinobacteria, albeit in lower proportions, in the gut of diverse triatomine species [21,22]. Nevertheless, it is well recognized that culture-based methods fail to describe the total diversity of natural bacterial communities. For example, intracellular symbionts such as *Wolbachia, Rohrkolberia* and some species of *Arsenophonus* that have been reported in triatomine guts [26,92,93] do not grow on artificial media. However, culture independent methods may also have specific biases that are still under evaluation.

DGGE is a technology that involves electrophoresis between the PCR amplification step and the qualitative visualization of amplicons. Therefore, DGGE can be applied to the screening and clustering of a larger number of individual samples, such as the gut microbiota of insects. Additionally, the fingerprint description of microbiota can be complemented by the extraction of sequence information from individual bands by their individual sequencing. However, the quantitative description of microbiota by DGGE is expected to lack precision because the relationship between band intensity and DNA quantity follows a logarithmic function. By contrast, the characterization of a 16S rDNA community is expected to be much more precise by 454 pyrosequencing because the bias in PCR amplification is expected to be the same as that of DGGE, but the quantitative response is proportional to the number of reads.

One of the few relevant differences in the results obtained by using DGGE-Sanger or pyrosequencing is that the less dominant non-actinobacteria genera detected by pyrosequencing in 35Tb and 19Tp samples were not observed by DGGE-Sanger or 16S rRNA random cloning and sequencing. Several non-mutually exclusive explanations can be considered for this discrepancy. First, the amplification of 16S rRNA genes was obtained using several universal primers. The different hypervariable V regions chosen for amplification can greatly influence the assessment of microbial diversity [94,95]. The V3-V1 region used for pyrosequencing seems to be taxonomically more informative than the V6-V8 region (with the use of F968-GC and R1401 primers used to generate the DGGE profiles) [27,94]. On the other hand, the V6-V8 region has been widely used in many DGGE studies because it produces higher quality DGGE profiles compared to the V3-V1 region, which produces poor quality profiles on DGGE with smeared bands [94].

An important feature of the process of full 16S rRNA amplification, cloning and posterior Sanger sequencing is that it allows the assembling of full-length 16S rDNA sequences (approximately 1.5 Kb) that most of the time allows the bacterial classification at the species level. The partial sequences of 16S rDNA bands separated by DGGE (approximately 430 bp) or obtained by 454 pyrosequencing (approximately 400 bp) only allowed the bacterial classification at the genus level.

## Conclusions

Overall, we demonstrate that the different culture independent analyses used here yielded similar albeit not identical results concerning the microbiota of *T. brasiliensis* and *T. pseudomaculata*. These microbiota are composed predominantly of *S. marcescens* and species of Actinobacteria of the suborder Corynebacterianeae. A high infection rate by different *T. cruzi* subpopulations was found in *Triatoma* specimens collected in peridomestic habitats. The bacterial microbiota in the *Triatoma* gut reared in the insectary can change over time when compared to field specimens. No clear correlation was found between *Triatoma* species, bacterial microbiota and *T. cruzi* infection in peridomestic habitats.

The high percentage of *T. cruzi* infection that was observed in *T. brasiliensis* and *T. pseudomaculata* should also be carefully considered by health authorities, as these triatomines are found in the peridomestic ecotopes of Russas and represent a risk of Chagas disease transmission to humans. Further research focusing on the role of the microbiota in the interaction with the parasite should be explored in more detail, as it is critically important to establish whether certain bacteria can prevent *T. cruzi* development in triatomines.

## Additional file

Additional file 1:
**Estimators of species diversity and richness for the sequenced samples.**
