# Supplementary material for: Characterization of the microbiota in the guts of Triatoma brasiliensis and Triatoma pseudomaculata infected by Trypanosoma cruzi in natural conditions using culture independent methods
Source: Parasit Vectors. 2015 Apr 24;8:245. doi: 10.1186/s13071-015-0836-z (PMC4429471; doi:10.1186/s13071-015-0836-z)
Supplement: Additional file 1: — Estimators of species diversity and richness for the sequenced samples. [file 13071_2015_836_MOESM1_ESM.doc]

**Additional file 1.** **Estimators of species diversity and richness for the sequenced samples**.

| Samples | N | Clusters | chao | H’ | Good | E |
| --- | --- | --- | --- | --- | --- | --- |
| 25Tb | 7,763 | 17 | 20.8 | 75.1 | 99.78 | 26.5 |
| 35Tb | 12,293 | 29 | 59.0 | 92.2 | 99.76 | 27.4 |
| 17Tp | 7,464 | 23 | 30.2 | 122.3 | 99.69 | 38.9 |
| 19Tp | 5,060 | 35 | 41.6 | 25.9 | 99.31 | 7.3 |

N: Number of sequences analyzed; Number of clusters observed for each distance (%) related to genus OTU 0.05; Chao: Chao1 estimator; H’: Shannon index; Good: Good’s coverage estimator; E: Equitability.
